# Colotomy

**Published:** 1889-12-28

**Authors:** 


### COLOTOMY.

The Bradshaw lecture on "Colotomy, Lumbago, and Iliac," by Mr. Bryant, vice-president of the Royal College of Surgeons, delivered December 5, demands attention. The subject was considered chiefly with reference to the different operations which should be adopted. While colotomy has been sanctioned, it has generally been regarded as a *dernier ressort* for the relief of chronic intestinal obstruction. Within the last decade, however, there has been a remarkable advance in the position of colotomy as an operation for the relief of rectal structure. There are two operations—the iliac or intra-peritoneal, and the lumbar or extra-peritoneal. The iliac operation is said to be easier than the lumbar; that, by means of the abdominal incision, the diagnosis in obscure cases may be verified before the bowel is opened; that there can be no probability of the surgeon mistaking the small intestines for the large; that the bowel can be more readily drawn out and fixed; that the inguinal position of the wound is more convenient to the patient. Mr. Bryant, examining these claims seriatim, shows, as we think, they are baseless. Moreover, there are certain cases of rectal cancer in which iliac colotomy seems quite inapplicable—that is, when the disease is high up and invades the bowel above the brim of the pelvis. Mr. Bryant has performed similar colotomy in more than 170 cases: 100 were urgent cases, and the operation was performed to ward off impending death. Of these 55 were successful, and 45 sank within the month; 70 cases were operated on before obstruction threatened life, and with the object of giving relief to the continual effort to pass stool, and to the pain associated with it. Not one of these cases sank from the operation. All convalesced, and expressed themselves grateful for the relief afforded. Eight survived the operation from two to six years. Mr. Bryant considers this operation is called for as soon as the first symptoms of impassable blockage appear in cancerous disease, for the malady makes slower progress as soon as the irritation caused by the fœces is removed. Mr. Bryant also gives some information as to the manner in which he performs lumbar colotomy. He always employs an oblique incision from above downwards, which follows the course of the nerves and vessels, allows room for the necessary manipulation, and falls naturally into the fold of skin above the crest of the ilium, which is constant in all, and well marked in fat people. This fold, Mr. Bryant believes, acts beneficial in preventing the prolapse of the bowel which surgeons employing other lines of incision complain of. At the same time it tends to keep the artificial anus closed. When the bowel is exposed it is separated with the fingers from its upper and lower attachments and allowed to bulge. It is then fixed. When the symptoms are urgent the bowel is opened at once. When there is no urgency the bowel is left *in situ* for two or three days.

